# Supplementary material for: Kaempferol as a Dietary Anti-Inflammatory Agent: Current Therapeutic Standing
Source: Molecules. 2020 Sep 7;25(18):4073. doi: 10.3390/molecules25184073 (PMC7570692; doi:10.3390/molecules25184073)
Supplement: Supplementary file 1 [file molecules-25-04073-s001.pdf]

Supplementary Table 1. Botanical sources of kaempferol/glycosides.

Species	Family	Kaempferol/glycosides	References
Abutilon theophrasti	Malvaceae	kaempferol 3-O-β-glucopyranoside, kaempferol 7-O-β-diglucoside	[1]
Acaenasplendens	Rosaceae	7-O-acetyl-3-O-β-D-glucosyl-kaempferol	[2]
Aceriphyllumrossii	Saxifragaceae	kaempferol 3-O-α-L-rhamnopyranosyl-(1→6)-β-D-glucopyranoside, kaempferol	[3]
Acacia nilotica	Leguminosae	Kaempferol	[4]
Aconitum spp	Ranunculaceae	kaempferol 3-O-(6-trans-p-coumaroyl)-β-glucopyranosyl-(12)-β-glucopyranoside-7-O-α-rhamnopyranoside, kaempferol 7-O-(6-trans-p-coumaroyl)-β-glucopyranosyl-(13)-α-rhamnopyranoside-3-O-β-glucopyranoside	[5]
Aconitum paniculatum	Ranunculaceae	Kaempferol 3-O-β-(2' '-acetyl)galactopyranoside	[6]
Actinidia valvata	Actinidiaceae	Kaempferol, kaempferol 3-O-β-D-galactopyranoside, kaempferol 3-O-α-L-rhamnopyranosyl-(1→3)-(4-O-acetyl-α-L-rhamnopyranosyl)-(1→6)-β-D-galactopyranoside.	[7]
Aconitum napellus	Ranunculaceae	kaempferol 7-O-(6-trans-p-coumaroyl)-β-glucopyranosyl-(13)-α-rhamnopyranoside-3-O-β-glucopyranoside	[8]
Adina racemosa	Rubiaceae	Kaempferol 3-O-α-L –rhamnopyranosyl-(1→6)-[(4-O-trans-p-coumaroyl)-α-L -rhamnopyranosyl (1→2)]-(4-O-trans-p-coumaroyl)-β-D-galactopyranoside	[9]
Allium cepa	Alliaceae	Kaempferol	[10]
Allium porrum	Alliaceae	Kaempferol 3-O-[2-O-(trans-3-methoxy-4-hydroxycinnamoyl)-β-D-galactopyranosyl]-(1→4)-O-β-D-glucopyranoside, kaempferol glycosides	[11]
Aloe vera	Asphodelaceae	Kaempferol	[12]
Althaea rosea	Malvaceae	Kaempferol	[13]
Argyreiaspeciosa	Convolvulaceae	Kaempferol 7-O methyl 3-sulphate	[14]
Apocynumvenetum	Apocynaceae	kaempferol 6'-O-acetate	[15,16]
Astragalus caprinus	Leguminosae	Kaempferol 3-O-[[β-D-xylopyranosyl(1→3)-α-L-rhamnopyranosyl(1→6)][β-D-apiofuranosyl(1→2)]]-β-D-galactopyranosyl	[17,18]
Baseonemaacuminatum	Asclepiadaceae	Kaempferol 3-O-(6''-galloyl)-β-D-glucopyranoside	[19]

Bauhinia malabarica	Leguminosae	Kaempferol and 6,8-di-C-methylkaempferol 3-methylether	[20]
Bauhinia microstachya	Leguminosae	Kaempferol 3-O-rhamnosyl	[21]
Berchemia floribunda	Rhamnaceae	Kaempferol and kaempferol 3-O-α-L-arabinofuranoside	[22]
Brassica campestris	Brassicaceae	Kaempferol and kaempferol 3-O-hydroxyferuloylsophoroside-7-O-glucoside	[23]
Bunias orientalis	Brassicaceae	kaempferol glycosides	[24]
Bupleurum flavum	Apiaceae	Kaempferol	[25]
Callistemon lanceolatus	Myrtaceae	Kaempferol 3-O-β-D-galacturonopyranoside	[26]
Campanula alliariifolia	Campanulaceae	Kaempferol 3-O-glucoside	[27]
Canavalia gladiate	Leguminosae	Kaempferol 3-O-β-D-galactopyranosyl-7-O-α-L-rhamnopyranoside	[28]
Carthamuslanatus	Asteraceae	Kaempferol 3-O-β-D-sophoroside	[29]
Cannabis sativa	Cannabaceae	Kaempferol 3-O-sophoroside	[30]
Cassia alata	Leguminosae	Kaempferol 3-O-gentiobioside	[31,32]
Cassia siamea	Leguminosae	Kaempferol	[33]
Chuquiraga spinosa	Asteraceae	Kaemperol 3-O-glucuronide, kaemperol 3-O-rutinoside and kaempherol 3-O-glucoside	[34]
Cinnamomumosmophloeum	Lauraceae	kaempferol 3-O-β-D-apiofuranosyl-(1→2)-α-L-arabinofuranosyl-7-O-α-L-rhamnopyranoside, Kaempferol 3-O-β-D-glucopyranosyl-(1→4)-α-L-rhamnopyranosyl-7-O-α-L-rhamnopyranoside	[35]
Citrus aurantifolia	Rutaceae	kaempferol 3-O-β-D-glucopyranoside-6''-(3-hydroxy-3-methyl glutarate), Kaempferol 3-O-β-rutinoside	[36]
Cuscutaaustralis	Convolvulaceae	Kaempferol	[37]
Datura suaveolens	Solanaceae	Kaempferol 3-O-α-L-arabinopyranosyl-7-O-β-D-glucopyranoside and kaempferol 3-O-α-L-arabinopyranoside	[38]
Dendrophthoe falcate	Loranthaceae	Kaempferol 3-O-α-L-rhamnopyranoside	[39]
Diodia teres	Rubiaceae	Kaempferol 3-O-rutinoside	[40]
Dipladenia martiana	Apocynaceae	Kaempferol	[41]
Dorycnium rectum	Leguminosae	Kaempferol-3,7-O-α-di-rhamnopyranoside	[42]
Dryopteris crassirhizoma	Aspidiaceae	Kaempferol glycosides	[43]
Drabanemorosa	Brassicaceae	Kaempferol glycosides	[44]
Echites hirsute	Apocynaceae	Kaempferol	[45]
Equisetum arvense	Equisetaceae	Kaempferol 3-O-glucoside	[46]
Equisetum myriochaetum	Equisetaceae	Kaempferol glucosides	[47]
Eruca sativa	Brassicaceae	Kaempferol glucosides	[48]
Eucalyptus occidentalis	Myrtaceae	6,8-di-C-methylkaempferol 3-methyl	[49]

		ether	
Euphorbia petiolata	Euphorbiaceae	kaempferol 3-O-glucoside and kaempferol 3-O-rhamnoside	[50]
Fagonia Arabica	Zygophyllaceae	Kaempferol 7-O-rhamnoside	[51]
Ficariaverna	Ranunculaceae	Kaempferol 3-O-β-D-(6''--L-rhamnopyranosyl)-glucopyranoside	[52]
Foeniculum vulgare	Apiaceae	kaempferol 3-O-glucoside and Kaempferol 3-O-rutinoside	[53]
Frankenia laevis	Frankeniaceae	Kaempferol 3,7-di-sodium sulphate	[54]
Geranium bellum	Geraniaceae	Kaempferol 3-O-β-D-glucopyranoside	[55]
Ginkgo biloba	Ginkgoaceae	kaempferol 3-O-α-(6'''-p-coumaroylglucosyl-β-1,4-rhamnoside), kaempferol 3-O-(2''-O-β-D-glucopyranosyl)-α-L-rhamnopyranoside	[56,57]
Glycyrrhiza spp	Leguminosae	Kaempferol 3-O-methyl ether	[58]
Grindelia robusta	Asteraceae	6-OH-kaempferol-3,6-dimethylether	[59]
Hedyotisdiffusa	Rubiaceae	kaempferol 3-O-[2-O-(6-O-E-feruloyl)-β-D-glucopyranosyl]-β-D-galactopyranoside	[60]
Hippophaerhamnoides	Elaeagnaceae	Kaempferol	[61,62]
Hydrangea macrophylla	Hydrangeaceae	Kaempferol oligoglycosides	[62]
Impatiens textori	Balsaminaceae	kaempferol 3-glucoside and kaempferol 3-rhamnosyldiglucoside	[63]
Indigofera suffruticosa	Leguminosae	Kaempferol	[64]
Ixeridium gracile	Asteraceae	Kaempferol	[65]
Kanahialaniflora	Asclepiadaceae	kaempferol 3-O-(2,6-di-O-α-L-rhamnopyranosyl)-β-D-glucopyranoside	[66]
Koelreuteriapaniculata	Sapindaceae	Kaempferol 3-O-arabinopyranoside	[67]
Lamium album	Lamiaceae	Kaempferol 3-O-glucoside	[68]
Laurus nobilis	Lauraceae	kaempferol 3-O-α-L-(2''-Z-p-coumaroyl-4''-E-p-coumaroyl)-rhamnoside	[69]
Leonurus persicus	Lamiaceae	Kaempferol 3-O-glucoside	[70]
Licanialicaniaeflora	Chrysobalanaceae	Kaempferol 3-O-α-rhamnoside	[71]
Lonicera japonica	Caprifoliaceae	Kaempferol 3-O-β-D-glucopyranoside	[72]
Machilusphilippinensis	Lauraceae	kaempferol 3-O-α-L-rhamnopyranoside 3''-E,4''-Z-di-p-coumaroic acid ester	[73]
Malva crispa	Malvaceae	Kaempferol glycosides	[74]
Meconopsisspp	Papaveraceae	kaempferol 3-O-(6-O-β-D-glucopyranosyl)-β-D-galactopyranoside	[75]
Melastomamalabathricum	Melastomataceae	Kaempferol 3-O-(2'',6''-di-O-p-trans-coumaroyl)-β-glucoside	[76]
Mitracarpusscaber	Rubiaceae	Kaempferol 3-O-rutinoside	[77]
Morindacitrifolia	Rubiaceae	Kaempferol	[78]
Neocheiropterispalmatopedata	Polypodiaceae	Kaempferol glycosides	[7]
Nepheliumlappaceum	Sapindaceae	Kaempferol 3-O-β-D-glucopyranoside-7-	[79]

		O-α-L-rhamnopyranoside	
Nicotiana tabacum	Solanaceae	Kaempferol 3-rutinoside	[80]
Ochnabeddomei	Ochnaceae	kaempferol 3-O-rhamnoside and kaempferol 3-O-glucoside	[81]
Olea europaea	Oleaceae	Kaempferol	[82]
Origanumdictamnus	Lamiaceae	Kaempferol	[83]
Oxytropis falcate	Leguminosae	Kaempferol	[84]
Papaver nudicaule	Papaveraceae	kaempferol 3-O-β-sophoroside-7-O-β-glucoside and other kaempferol derivatives	[85]
Pedilanthustithymaloides	Euphorbiaceae	Kaempferol 3-O-β-D-glucopyranoside-6''-(3-hydroxy-3-methylglutarate)	[86]
Peumusboldus	Monimiaceae	Kaempferol glycosides	[87]
Phellodendronamurense	Rutaceae	Kaempferol 3-O-β-D-glucoside	[88]
Phlomiscaucasica	Lamiaceae	Kaempferol 3-O-glucoside	[89]
Pinus densiflora	Pinaceae	Kaempferol 3-O-β-D-glucoside and 6-C-methyl kaempferol 3-O-β-D-glucoside	[90]
Pisum sativum	Leguminosae	Kaempferol 3-sophorotrioside	[91]
Polygala japonica	Polygalaceae	Kaempferol 3-gentiobioside	[92]
Prunus amygdalus	Rosaceae	Kaempferol 3-O-α-L-rhamnopyranosyl-(1→6)-β-D-glucopyranoside	[93]
Prunus serrulata	Rosaceae	kaempferol 3-O-β-xylopyranoside, kaempferol 3-O-β-glucopyranoside	[94]
Quercus dentate	Fagaceae	kaempferol 3-O-(6''-trans-p-coumaroyl)-β-D-glucopyranoside, kaempferol 3-O-(2'',4''-di-acetyl-3''-cis-p-coumaroyl-6''-trans-p-coumaroyl)-β-D-glucopyranoside	[95]
Randia Formosa	Rubiaceae	Kaempferol 3-O-rutinoside	[96]
Rhamnusnipalensis	Rhamnaceae	Kaempferol 4'-methylether	[97]
Rhamnusprocumbens	Rhamnaceae	Kaempferol	[98]
Rosa canina	Rosaceae	Kaempferol 3-O-β-D-(6''-E-p-coumaroyl)-glucopyranoside	[99]
Rosmarinus officinalis	Lamiaceae	Kaempferol	[100]
Rubussanctus	Rosaceae	kaempferol 3-O-β-D-galactosides , kaempferol 3-O-β-L-arabinopyranoside	[101]
Sageretiatheezans	Rhamnaceae	Kaempferol 3-O-α-L-rhamnopyranoside	[102]
Sambucus nigra	Caprifoliaceae	Kaempferol	[103]
Scabiosa hymettia	Dipsacaceae	kaempferol 3-O-β-D-glucopyranoside, Kaempferol 3-O-[3- O-acetyl-6-O-(E)-p-coumaroyl]-β-D-glucopyranoside	[104]
Scopoliacaucasica	Solanaceae	kaempferol 3-O-(2-glucosyl)-galactoside	[105]
Scopolialurida	Solanaceae	Kaempferol glycosides	[106]
Scrophulariailwensis	Scrophulariaceae	Kaempferol 3-O-rutinoside	[107]
Senecio scandens	Asteraceae	Kampferol 3-O-rhamnoside	[108]
Solanum nigrum	Solanaceae	Kaempferol	[109]
Solidagoaltissima	Asteraceae	Kaempferol 3-O-β-D-apiofuranosyl-(1→6)-β-D-glucopyranoside	[110]

Spiraea canescens	Rosaceae	6'-O-(4''-methoxy-trans-cinnamoyl)-kaempferol-3-β-D-glucopyranoside	[111]
Syzygiumaromaticum	Myrtaceae	Kaempferol	[112]
Tamarixnilotica	Tamaricaceae	Kaempferol 4'-methyl ether	[113]
Terminalia myriocarpa	Combretaceae	Kaempferol 3-O-β-D-rutinoside	[114]
Thevetia peruviana	Apocynaceae	Kaempferol 3-glucosyl-(1→4)-[6'''-sinapoylglucosyl]-(1→2)-galactoside	[115]
Trifoliumalexandrinum	Leguminosae	Kaempferol	[116]
Trigonella foenum-graecum	Leguminosae	kaempferol 3-O-β-D-glucosyl-(1→2)-(6''-O-acetyl)-β-D-galactoside-7-O-β-D-glucoside	[117]
Ullucustuberosus	Basellaceae	Kaempferol 3-O-(2'',6''-di-O-α-L-rhamnopyranosil)-β-D-glucopyranoside	[118]
Vaccinium vitis-idaea	Ericaceae	kaempferol 3-O-[4''-(3-hydroxy-3-methylglutaroyl)]-α-rhamnose, Kaempferol-pentoside	[119]
Vahliacapensis	Vahliaceae	Kaempferol	[120]
Vernonia ferruginea	Asteraceae	Kaempferol 3-O-β-D-apiofuranosyl-(1→4)-β-D-glucopyranoside	[121]
Vernonia travancorica	Asteraceae	4'-methoxykaempferol, Kaempferol 3-O-β-[β-(6'''-acetyl)-D-glucopyranosyl-(1→2)]-D-glucopyranosyl	[122]
Vinca minor	Apocynaceae	Kaempferol glycosides	[123]
Vitis vinifera	Vitaceae	kaempferol 3-glucoside, kaempferol 3-glucuronide	[124]
Waltheriaindica	Sterculiaceae	Kaempferol 3-O-β-D-(6''-O-coumaroyl)glucopyranoside	[125]
Warburgiaugandensis	Canellaceae	kaempferol 3-rhamnoside,kaempferol 3-glucoside, kaempferol 3-arabinoside,Kaempferol 3-O-α-rhamnoside-7,4'-di-O-β-galactoside	[126]
Zelkova oregoniana	Ulmaceae	Kaempferol	[127]
Zollerniailicifolia	Leguminosae	Kaempferol 3-O-α-L-rhamnopyranosyl-(1→2)-O-[α-L-rhamnopyranosyl-(1→6)]-O-β-D-galactopyranoside-7-O-α-L-rhamnopyranoside	[128]

References

1. Matławska, I.; Sikorska, M. Flavonoids from Abutilon theophrasti flowers. *Acta Pol. Pharm.* 2005, *62*, 135–139.
2. Backhouse, N.; Delporte, C.; Negrete, R.; Feliciano, S.S.; López-Pérez, J. Bioactive phenolic derivatives from Acaenasplendens methanol extract. *Phytother. Res.* **2002**, *16*, 562–566.
3. Han, J.-T.; Bang, M.-H.; Chun, O.-K.; Kim, D.-O.; Lee, C.-Y.; Baek, N.-I. Flavonol glycosides from the aerial parts of Aceriphyllumrossii and their antioxidant activities. *Arch. Pharmacal Res.* **2004**, *27*, 390–395.
4. Singh, R.; Singh, B.; Singh, S.; Kumar, N.; Kumar, S.; Arora, S. Anti-free radical activities of kaempferol isolated from Acacia nilotica (L.) Willd. Ex. Del. *Toxicol. Vitr.***2008**, *22*, 1965–1970, doi:10.1016/j.tiv.2008.08.007.
5. Braca, A.; Fico, G.; Morelli, I.; De Simone, F.; Tomè, F.; De Tommasi, N. Antioxidant and free radical scavenging activity of flavonol glycosides from different Aconitum species. *J. Ethnopharmacol.***2003**, *86*, 63–67, doi:10.1016/s0378-8741(03)00043-6.
6. Fico, G.; Braca, A.; Bilia, A.R.; Tomè, F.; Morelli, I. Flavonol Glycosides from the Flowers of Aconitumpaniculatum. *J. Nat. Prod.***2000**, *63*, 1563–1565, doi:10.1021/np000246h.
7. Xin, H.L.; Wu, Y.C.; Su, Y.H.; Sheng, J.Y.; Ling, C.Q. Novel Flavonoids from the Leaves of Actinidia valvata Dunn: Structural Elucidation and Antioxidant Activity. *Planta Med.* **2011**, *77*, 70–73, doi:10.1055/s-0030-1250113.
8. Fico, G.; Braca, A.; De Tommasi, N.; Tomè, F.; Morelli, I. Flavonoids from Aconitum napellus subsp. neomontanum. *Phytochemistry***2001**, *57*, 543–546, doi:10.1016/s0031-9422(01)00102-9.
9. Itoh, A.; Tanahashi, T.; Nagakura, N.; Takenaka, Y.; Chen, C.-C.; Pelletier, J. Flavonoid Glycosides fromAdinaracemosaand Their Inhibitory Activities on Eukaryotic Protein Synthesis. *J. Nat. Prod.***2004**, *67*, 427–431, doi:10.1021/np030440e.
10. Rodríguez Galdón, B.; Rodríguez Rodríguez, E.M.; Díaz Romero, C. Flavonoids in Onion Cultivars (Allium cepaL.). *J. Food Sci.***2008**, *73*, C599–C605, doi:10.1111/j.1750-3841.2008.00903.x.
11. Fattorusso, E.; Lanzotti, V.; Taglialatela-Scafati, O.; Cicala, C. The flavonoids of leek, Allium porrum. *Phytochemistry***2001**, *57*, 565–569, doi:10.1016/s0031-9422(01)00039-5.
12. Keyhanian, S.; Stahl-Biskup, E. Phenolic Constituents in Dried Flowers of Aloe vera (Aloe barbadensis) and their in vitro Antioxidative Capacity. *Planta Med.* **2007**, *73*, 599–602, doi:10.1055/s-2007-967202.
13. Papiez, M.; Gancarczyk, M.; Bilińska, B. The compounds from the hollyhock extract (Althaea rosea Cav. var. nigra) affect the aromatization in rat testicular cells in vivo and in vitro. *Folia Histochem. Cytobiol.***2002**, *40*, 353–359.
14. Habbu, P.; Mahadevan, K.; Shastry, R.; Manjunatha, H. Antimicrobial activity of flavanoid sulphates and other fractions of Argyreiaspeciosa (Burm. f) Boj. *Indian J. Exp. Biol.* **2009**, *47*, 121–128.
15. Grundmann, O.; Nakajima, J.-I.; Kamata, K.; Seo, S.; Butterweck, V. Kaempferol from the leaves of Apocynumvenetum possesses anxiolytic activities in the elevated plus maze test in mice. *Phytomedicine***2009**, *16*, 295–302.
16. Xiong, Q.; Fan, W.; Tezuka, Y.; Adnyana, I.K.; Stampoulis, P.; Hattori, M.; Namba, T.; Kadota, S. Hepatoprotective effect of Apocynumvenetum and its active constituents. *Planta Med.* **2000**, *66*, 127–133.
17. Semmar, N.; Fenet, B.; Lacaille-Dubois, M.-A.; Gluchoff-Fiasson, K.; Chemli, R.; Jay, M. Two New Glycosides fromAstragaluscaprinus. *J. Nat. Prod.***2001**, *64*, 656–658, doi:10.1021/np0005311.
18. Semmar, N.; Fenet, B.; Gluchoff-Fiasson, K.; Hasan, A.; Jay, M. Four New Flavonol Glycosides from the Leaves of Astragalus c aprinus. *J. Nat. Prod.***2002**, *65*, 576–579.
19. De Leo, M.; Braca, A.; De Tommasi, N.; Norscia, I.; Morelli, I.; Battinelli, L.; Mazzanti, G. Phenolic compounds from Baseonemaacuminatum leaves: Isolation and antimicrobial activity. *Planta Med.* **2004**, *70*, 841–846.
20. Kaewamatawong, R.; Kitajima, M.; Kogure, N.; Takayama, H. Flavonols from Bauhinia malabarica. *J. Nat. Med.***2008**, *62*, 364–365, doi:10.1007/s11418-008-0249-9.
21. Meyre-Silva, C.; Yunes, R.A.; Monache, F.; Santos, A.; Schmeling, L.O.; de Maria Gadotti, V.; Liz, F.; Cechinel-Filho, V. Phytochemical and pharmacological analysis of Bauhinia microstachya (Raddi) Macbr. (Leguminosae). *Z. Nat. C***2001**, *56*, 939–942.
22. Wang, Y.F.; Cao, J.X.; Efferth, T.; Lai, G.F.; Luo, S.D. Cytotoxic and New Tetralone Derivatives from Berchemia floribunda (Wall.) Brongn. *Chem. Biodivers.***2006**, *3*, 646–653, doi:10.1002/cbdv.200690067.

23. Harbaum, B.; Hubbermann, E.M.; Wolff, C.; Herges, R.; Zhu, Z.; Schwarz, K. Identification of Flavonoids and Hydroxycinnamic Acids in Pak Choi Varieties (Brassica campestrisL. ssp.chinensisvar.communis) by HPLC–ESI-MSnand NMR and Their Quantification by HPLC–DAD. *J. Agric. Food Chem.***2007**, *55*, 8251–8260, doi:10.1021/jf071314+.
24. Bennett, R.N.; Rosa, E.A.; Mellon, F.A.; Kroon, P.A. Ontogenic profiling of glucosinolates, flavonoids, and other secondary metabolites in Eruca sativa (salad rocket), Diplotaxiserucoides (wall rocket), Diplotaxistenuifolia (wild rocket), and Bunias orientalis (Turkish rocket). *J. Agric. Food Chem.* **2006**, *54*, 4005–4015.
25. Pistelli, L.; Noccioli, C.; Giachi, I.; Dimitrova, B.; Gevrenova, R.; Morelli, I.; Potenza, D. Lupane-triterpenes from Bupleurum flavum. *Nat. Prod. Res.***2005**, *19*, 783–788, doi:10.1080/14786410500045119.
26. Mahmoud, I.; Moharram, F.; Marzouk, M.; Linscheid, M.; Saleh, M. Polyphenolic constituents of Callistemon lanceolatus leaves. *Die Pharm.***2002**, *57*, 494–496.
27. Dumlu, M.U.; Gurkan, E.; Tuzlaci, E. Chemical composition and antioxidant activity of Campanula alliariifolia. *Nat. Prod. Res.***2008**, *22*, 477–482, doi:10.1080/14786410701640429.
28. Murakami, T.; Kohno, K.; Kishi, A.; Matsuda, H.; Yoshikawa, M. Medicinal foodstuffs. XIX. Absolute stereostructures of canavalioside, a new Ent-kaurane-type diterpene glycoside, and gladiatosides A1, A2, A3, B1, B2, B3, C1, and C2, new acylated flavonol glycosides, from sword bean, the seeds of Canavalia gladiata. *Chem. Pharm. Bull.***2000**, *48*, 1673–1680, doi:10.1248/cpb.48.1673.
29. Taskova, R.; Mitova, M.; Mikhova, B.; Duddeck, H. Bioactive phenolics from Carthamuslanatus, L.Z. *Nat. C***2003**, *58*, 704–707.
30. Ross, S.A.; ElSohly, M.A.; Sultana, G.N.; Mehmedic, Z.; Hossain, C.F.; Chandra, S. Flavonoid glycosides and cannabinoids from the pollen of Cannabis sativa L. *Phytochem. Anal.***2005**, *16*, 45–48, doi:10.1002/pca.809.
31. Moriyama, H.; Iizuka, T.; Nagai, M. A Stabilized Flavonoid Glycoside in Heat-Treated Cassia alata Leaves and Its Structural Elucidation. *YakugakuZasshi***2001**, *121*, 817–820, doi:10.1248/yakushi.121.817.
32. Moriyama, H.; Iizuka, T.; Nagai, M.; Miyataka, H.; Satoh, T. Antiinflammatory activity of heat-treated Cassia alata leaf extract and its flavonoid glycoside. *YakugakuZasshi***2003**, *123*, 607–611, doi:10.1248/yakushi.123.607.
33. Ntandou, G.N.; Banzouzi, J.; Mbatchi, B.; Elion-Itou, R.; Etou-Ossibi, A.; Ramos, S.; Benoit-Vical, F.; Abena, A.; Ouamba, J. Analgesic and anti-inflammatory effects of Cassia siamea Lam. stem bark extracts. *J. Ethnopharmacol.***2010**, *127*, 108–111, doi:10.1016/j.jep.2009.09.040.
34. Landa, A.; Casado, R.; Calvo, M.I. Identification and quantification of flavonoids from Chuquiraga spinosa (Asteraceae). *Nat. Prod. Commun.***2009**, *4*, 1934578X0900401008, doi:10.1177/1934578x0900401008.
35. Fang, S.-H.; Rao, Y.K.; Tzeng, Y.-M. Inhibitory effects of flavonol glycosides from Cinnamomumosmophloeum on inflammatory mediators in LPS/IFN-γ-activated murine macrophages. *Bioorg. Med. Chem.* **2005**, *13*, 2381–2388.
36. Berhow, M.A.; Bennett, R.D.; Poling, S.M.; Vannier, S.; Hidaka, T.; Omura, M. Acylated flavonoids in callus cultures of Citrus aurantifolia. *Phytochemistry***1994**, *36*, 1225–1227, doi:10.1016/s0031-9422(00)89641-7.
37. Ye, M.; Yan, Y.; Guo, D.-A. Characterization of phenolic compounds in the Chinese herbal drug Tu-Si-Zi by liquid chromatography coupled to electrospray ionization mass spectrometry. *Rapid Commun. Mass Spectrom.***2005**, *19*, 1469–1484, doi:10.1002/rcm.1944.
38. Sajeli Begum, A.; Sahai, M.; Fujimoto, Y.; Asai, K.; Schneider, K.; Nicholson, G.; Suessmuth, R. A new kaempferol diglycoside from Datura suaveolensHumb. &Bonpl. ex. Willd. *Nat. Prod. Res.* **2006**, *20*, 1231–1236.
39. Mallavadhani, U.V.; Narasimhan, K.; Sudhakar, A.V.S.; Mahapatra, A.; Li, W.; van Breemen, R.B. Three new pentacyclic triterpenes and some flavonoids from the fruits of an Indian Ayurvedic plant Dendrophthoe falcata and their estrogen receptor binding activity. *Chem. Pharm. Bull.* **2006**, *54*, 740–744.
40. Cordell, G.; Lyon, R.; Fong, H.; Benoit, P.; Farnsworth, N. Biological and phytochemical investigations of Dianthus barbatus cv. "China Doll" (Caryophyllaceae). *Lloydia* 1977, *40*, 361–363.
41. de Cranchi, M.G.; Carvalho Cranchi, D.; Kingston, D.G.; Werle, A.A. Proposed active constituents of Dipladenia martiana. *Phytother. Res.***2001**, *15*, 715–717, doi:10.1002/ptr.865.
42. Moreno, A.; Cordero, C.M.; Iglesias-Guerra, F.; Toro, M.V. Flavonoids from Dorycnium rectum. *Biochem. Syst. Ecol.***2002**, *30*, 73–74, doi:10.1016/s0305-1978(01)00070-9.

43. Min, B.-S.; Tomiyama, M.; Nakamura, N.; Hattori, M. Kaempferol Acetylrhamnosides from the Rhizome of Dryopteris crassirhizoma and Their Inhibitory Effects on Three Different Activities of Human Immunodeficiency Virus-1 Reverse Transcriptase. *Chem. Pharm. Bull.***2001**, *49*, 546–550, doi:10.1248/cpb.49.546.
44. Moon, S.-S.; Rahman, A.A.; Manir, M.; Ahamed, V.J. Kaempferol glycosides and cardenolide glycosides, cytotoxic constituents from the seeds of Drabanemorosa (Brassicaceae). *Arch. Pharmacal Res.* **2010**, *33*, 1169–1173.
45. Chien, M.; Svoboda, G.; Schiff, Jr, P.; Slatkin, D.; Knapp, J. Chemical constituents of Echiteshirsuta (Apocynaceae). *J. Pharm. Sci.* **1979**, *68*, 247–249.
46. Oh, H.; Kim, D.-H.; Cho, J.-H.; Kim, Y.-C. Hepatoprotective and free radical scavenging activities of phenolic petrosins and flavonoids isolated from Equisetum arvense. *J. Ethnopharmacol.***2004**, *95*, 421–424, doi:10.1016/j.jep.2004.08.015.
47. Cetto, A.A.; Wiedenfeld, H.; Revilla, M.C.; Sergio, I.A. Hypoglycemic effect of Equisetum myriochaetum aerial parts on streptozotocin diabetic rats. *J. Ethnopharmacol.***2000**, *72*, 129–133, doi:10.1016/s0378-8741(00)00218-x.
48. Benyahia, S.; Benayache, S.; Benayache, F.; Quintana, J.; López, M.; León, F.; Hernández, J.C.; Estévez, F.; Bermejo, J. Isolation from eucalyptus o ccidentalis and identification of a new Kaempferol derivative that induces apoptosis in human myeloid leukemia cells. *J. Nat. Prod.* **2004**, *67*, 527–531.
49. Lee, M.K.; Jeon, H.Y.; Lee, K.Y.; Kim, S.H.; Ma, C.J.; Sung, S.H.; Lee, H.-S.; Park, M.J.; Kim, Y. Inhibitory Constituents of Euscaphis japonica on Lipopolysaccharide-Induced Nitric Oxide Production in BV2 Microglia. *Planta Med.* **2007**, *73*, 782–786, doi:10.1055/s-2007-981551.
50. El-Wakil, E.A. Phytochemical and molluscicidal investigations of Fagonia arabica. *Z. Nat. C***2007**, *62*, 661–667, doi:10.1515/znc-2007-9-1006.
51. Tomczyk, M.; Gudej, J.; Sochacki, M. Flavonoids from FicariavernaHuds. *Z. Nat. C***2002**, *57*, 440–444.
52. Parejo, I.; Viladomat, F.; Bastida, J.; Schmeda-Hirschmann, G.; Burillo, J.; Codina, C. Bioguided Isolation and Identification of the Nonvolatile Antioxidant Compounds from Fennel (FoeniculumvulgareMill.) Waste. *J. Agric. Food Chem.***2004**, *52*, 1890–1897, doi:10.1021/jf030717g.
53. Hussein, S.A.M. Flavonoid and methoxyellagic acid sodium sulphates from Frankenia laevis L. *Die Pharm.* 2004, *59*, 484–487.
54. Gayosso-De-Lucio, J.A.; Torres-Valencia, J.M.; Cerda-García-Rojas, C.M.; Joseph-Nathan, P. Ellagitannins from Geranium potentillaefolium and G. bellum. *Nat. Prod. Commun.***2010**, *5*, 1934578X1000500407, doi:10.1177/1934578x1000500407.
55. Lee, H.-S.; Kim, M.-J. Selective responses of three Ginkgo biloba leaf-derived constituents on human intestinal bacteria. *J. Agric. Food Chem.***2002**, *50*, 1840–1844, doi:10.1021/jf011140a.
56. Kwon, S.H.; Nam, J.I.; Kim, S.H.; Kim, J.H.; Yoon, J.H.; Kim, K.S. Kaempferol and quercetin, essential ingredients in Ginkgo bilobaextract, inhibit interleukin-1β-induced MUC5AC gene expression in human airway epithelial cells. *Phytother. Res.***2009**, *23*, 1708–1712, doi:10.1002/ptr.2817.
57. Hatano, T.; Yasuhara, T.; Fukuda, T.; Noro, T.; Okuda, T. Phenolic constituents of licorice. II. Structures of licopyranocoumarin, licoarylcoumarin and glisoflavone, and inhibitory effects of licorice phenolics on xanthine oxidase. *Chem. Pharm. Bull.***1989**, *37*, 3005–3009, doi:10.1248/cpb.37.3005.
58. Krenn, L.; Wollenweber, E.; Steyrleuthner, K.; Görick, C.; Melzig, M.F. Contribution of methylated exudate flavonoids to the anti-inflammatory activity of Grindelia robusta. *Fitoterapia***2009**, *80*, 267–269, doi:10.1016/j.fitote.2009.03.001.
59. Kim, Y.; Park, E.J.; Kim, J.; Kim, Y.-B.; Kim, S.R.; Kim, Y.C. Neuroprotective constituents from Hedyotisdiffusa. *J. Nat. Prod.* **2001**, *64*, 75–78.
60. Sharma, U.K.; Sharma, K.; Sharma, N.; Sharma, A.; Singh, H.P.; Sinha, A.K. Microwave-assisted efficient extraction of different parts of Hippophaerhamnoides for the comparative evaluation of antioxidant activity and quantification of Its phenolic constituents by reverse-phase high-performance liquid chromatography (RP-HPLC). *J. Agric. Food Chem.***2008**, *56*, 374–379.
61. Yoshikawa, M.; Matsuda, H.; Shimoda, H.; Shimada, H.; Harada, E.; Naitoh, Y.; Miki, A.; Yamahara, J.; Murakami, N. Development of bioactive functions in hydrangeaedulcis folium. V. On the antiallergic and antimicrobial principles of hydrangeaedulcisfolium.(2). Thunberginols C, D, and E, thunberginol G 3′-O-

glucoside,(-)-hydrangenol 4′-o-glucoside, and (+)-hydrangenol 4′-O-glucoside. *Chem. Pharm. Bull.* **1996**, *44*, 1440–1447.
62. Ueda, Y.; Oku, H.; Iinuma, M.; Ishiguro, K. Effects on Blood Pressure Decrease in Response to PAF of Impatiens textori M IQ. *Biol. Pharm. Bull.* **2003**, *26*, 1505–1507.
63. Calvo, T.R.; Cardoso, C.R.P.; da Silva Moura, A.C.; dos Santos, L.C.; Colus, I.M.S.; Vilegas, W.; Varanda, E.A. Mutagenic activity of Indigoferatruxillensis and I. suffruticosa aerial parts. *Evid. Based Complement. Altern. Med.* **2011**, *2011*, 323276.
64. Zhang, Y.; Zhao, L.; Shi, Y.-P. Separation and determination of flavonoids in Ixeridium gracile by capillary electrophoresis. *J. Chromatogr. Sci.***2007**, *45*, 600–604, doi:10.1093/chromsci/45.9.600.
65. Clarkson, C.; Stærk, D.; Hansen, S.H.; Jaroszewski, J.W. Hyphenation of Solid-Phase Extraction with Liquid Chromatography and Nuclear Magnetic Resonance: Application of HPLC-DAD-SPE-NMR to Identification of Constituents of Kanahialaniflora. *Anal. Chem.***2005**, *77*, 3547–3553, doi:10.1021/ac050212k.
66. Lin, W.-H.; Deng, Z.-W.; Lei, H.-M.; Fu, H.-Z.; Li, J. Polyphenolic compounds from the leaves of KoelreuteriapaniculataLaxm. *J. Asian Nat. Prod. Res.* **2002**, *4*, 287–295.
67. Budzianowski, J.; Skrzypczak, L. Phenylpropanoid esters from Lamium album flowers. *Phytochemistry***1995**, *38*, 997–1001, doi:10.1016/0031-9422(94)00727-b.
68. Otsuka, N.; Liu, M.-H.; Shiota, S.; Ogawa, W.; Kuroda, T.; Hatano, T.; Tsuchiya, T. Anti-methicillin resistant Staphylococcus aureus (MRSA) compounds isolated from Laurus nobilis. *Biol. Pharm. Bull.***2008**, *31*, 1794–1797, doi:10.1248/bpb.31.1794.
69. Tasdemir, D.; Scapozza, L.; Zerbe, O.; Linden, A.; Calis, I.; Sticher, O. Iridoid Glycosides of Leonurus persicus. *J. Nat. Prod.***1999**, *62*, 811–816, doi:10.1021/np980376e.
70. Braca, A.; Sortino, C.; Politi, M.; Morelli, I.; Mendez, J. Antioxidant activity of flavonoids from Licanialicaniaeflora. *J. Ethnopharmacol.* **2002**, *79*, 379–381.
71. Choi, C.-W.; Jung, H.A.; Kang, S.S.; Choi, J.S. Antioxidant constituents and a new triterpenoid glycoside from FlosLonicerae. *Arch. Pharmacal Res.***2007**, *30*, 1–7, doi:10.1007/bf02977770.
72. Lee, S.-S.; Lin, H.-C.; Chen, C.-K. Acylated flavonolmonorhamnosides, α-glucosidase inhibitors, from Machilusphilippinensis. *Phytochemistry***2008**, *69*, 2347–2353.
73. Matławska, I.; Sikorska, M. Flavonoids from flowers of Malva crispa L. (Malvaceae). *Acta Pol. Pharm.* 2004, *61*, 65–68.
74. Tanaka, M.; Fujimori, T.; Uchida, I.; Yamaguchi, S.; Takeda, K. A malonylated anthocyanin and flavonols in blue Meconopsis flowers. *Phytochemistry***2001**, *56*, 373–376, doi:10.1016/s0031-9422(00)00357-5.
75. Sirat, H.M.; Susanti, D.; Ahmad, F.; Takayama, H.; Kitajima, M. Amides, triterpene and flavonoids from the leaves of Melastomamalabathricum L. *J. Nat. Med.* **2010**, *64*, 492–495.
76. Bisignano, G.; Sanogo, R.; Marino, A.; Aquino, R. ʻangelo, V.; Germano, M.; De Pasquale, R.; Pizza, C. Antimicrobial activity of Mitracarpusscaber extract and isolated constituents. *Lett. Appl. Microbiol.* **2000**, *30*, 105–108.
77. Kim, S.Y.; Gao, J.J.; Lee, W.-C.; Ryu, K.S.; Lee, K.R.; Kim, Y.C. Antioxidative flavonoids from the leaves of Morus alba. *Arch. Pharmacal Res.***1999**, *22*, 81–85, doi:10.1007/bf02976442.
78. Ragasa, C.Y.; de Luna, R.D.; Cruz, W.C.; Rideout, J.A. Monoterpene Lactones from the Seeds of Nephelium l appaceum. *J. Nat. Prod.* **2005**, *68*, 1394–1396.
79. Yang, J.-H.; Kondratyuk, T.P.; Marler, L.E.; Qiu, X.; Choi, Y.; Cao, H.; Yu, R.; Sturdy, M.; Pegan, S.; Liu, Y. Isolation and evaluation of kaempferol glycosides from the fern Neocheiropterispalmatopedata. *Phytochemistry***2010**, *71*, 641–647.
80. Pang, T.; Yuan, Z.; Dai, Y.; Wang, C.; Yang, J.; Peng, L.; Xu, G. Identification and determination of glycosides in tobacco leaves by liquid chromatography with atmospheric pressure chemical ionization tandem mass spectrometry. *J. Sep. Sci.***2007**, *30*, 289–296, doi:10.1002/jssc.200600236.
81. Jayaprakasam, B.; Damu, A.; Rao, K.; Gunasekar, D.; Blond, A.; Bodo, B. 7-O-Methyltetrahydroochnaflavone, a new biflavanone from Ochnabeddomei. *J. Nat. Prod.* **2000**, *63*, 507–508.

82. De Laurentis, N.; Stefanizzi, L.; Milillo, M.; Tantillo, G. Flavonoids from leaves of Olea europaea L. cultivars. *Ann. Pharm. Françaises***1998**, *56*, 268.
83. Chatzopoulou, A.; Karioti, A.; Gousiadou, C.; Lax Vivancos, V.; Kyriazopoulos, P.; Golegou, S.; Skaltsa, H. Depsides and other polar constituents from Origanumdictamnus L. and their in vitro antimicrobial activity in clinical strains. *J. Agric. Food Chem.* **2010**, *58*, 6064–6068.
84. Jiang, H.; Zhan, W.; Liu, X.; Jiang, S. Antioxidant activities of extracts and flavonoid compounds from Oxytropis falcate Bunge. *Nat. Prod. Res.***2008**, *22*, 1650–1656, doi:10.1080/14786410701875686.
85. Schliemann, W.; Schneider, B.; Wray, V.; Schmidt, J.; Nimtz, M.; Porzel, A.; Böhm, H. Flavonols and an indole alkaloid skeleton bearing identical acylated glycosidic groups from yellow petals of Papaver nudicaule. *Phytochemistry***2006**, *67*, 191–201, doi:10.1016/j.phytochem.2005.11.002.
86. Abreu, P.M.; Matthew, S.; González, T.; Vanickova, L.; Costa, D.; Gomes, A.; Segundo, M.A.; Fernandes, E. Isolation and identification of antioxidants from Pedilanthustithymaloides. *J. Nat. Med.* **2008**, *62*, 67–70.
87. Simirgiotis, M.J.; Schmeda-Hirschmann, G. Direct identification of phenolic constituents in Boldo Folium (Peumusboldus Mol.) infusions by high-performance liquid chromatography with diode array detection and electrospray ionization tandem mass spectrometry. *J. Chromatogr. A***2010**, *1217*, 443–449, doi:10.1016/j.chroma.2009.11.014.
88. Leu, C.-H.; Li, C.-Y.; Yao, X.; Wu, T.-S.; Constituents from the leaves of Phellodendronamurense and their antioxidant activity. *Chem. Pharm. Bull.* **2006**, *54*, 1308–1311.
89. Delazar, A.; Sabzevari, A.; Mojarrab, M.; Nazemiyeh, H.; Esnaashari, S.; Nahar, L.; Razavi, S.M.; Sarker, S.D. Free-radical-scavenging principles from Phlomiscaucasica. *J. Nat. Med.* **2008**, *62*, 464.
90. Jung, M.J.; Jung, H.A.; Kang, S.S.; Hwang, G.-S.; Choi, J.S. A new abietic acid-type diterpene glucoside from the needles of Pinus densiflora. *Arch. Pharmacal Res.***2009**, *32*, 1699–1704, doi:10.1007/s12272-009-2206-x.
91. Ferreres, F.; Esteban, E.;Carpena-Ruiz, R.; Jiménez, M.A.; Tomás-Barberán, F.A. Acylated flavonolsophorotriosides from pea shoots. *Phytochemistry***1995**, *39*, 1443–1446.
92. Kim, S.H.; Jang, S.-D.; Lee, K.Y.; Sung, S.H.; Kim, Y.C. Chemical constituents isolated from Polygala japonica leaves and their inhibitory effect on nitric oxide production in vitro. *J. Enzym. Inhib. Med. Chem.***2009**, *24*, 230–233, doi:10.1080/14756360802051362.
93. Sang, S.; Lapsley, K.; Jeong, W.-S.; Lachance, P.A.; Ho, C.-T.; Rosen, R.T. Antioxidative Phenolic Compounds Isolated from Almond Skins (Prunus amygdalusBatsch). *J. Agric. Food Chem.***2002**, *50*, 2459–2463, doi:10.1021/jf011533+.
94. Jung, H.A.; Kim, A.R.; Chung, H.Y.; Choi, J.S. In vitro antioxidant activity of some selectedprunus species in Korea. *Arch. Pharmacal Res.***2002**, *25*, 865–872, doi:10.1007/bf02977006.
95. Meng, Z.; Zhou, Y.; Lu, J.; Sugahara, K.; Xu, S.; Kodama, H. Effect of five flavonoid compounds isolated from Quercus dentata Thunb on superoxide generation in human neutrophils and phosphorylation of neutrophil proteins. *Clin. Chim. Acta***2001**, *306*, 97–102, doi:10.1016/s0009-8981(01)00403-x.
96. Sahpaz, S.; Gupta, M.P.; Hostettmann, K. Triterpene saponins from Randiaformosa. *Phytochemistry***2000**, *54*, 77–84.
97. Singh, S.; Pandey, M.; Singh, A.; Singh, U.; Pandey, V. A new chalcone glycoside from Rhamnusnipalensis. *Nat. Prod. Res.* **2008**, *22*, 1657–1659.
98. Schieber, A.; Mihalev, K.; Berardini, N.; Mollov, P.; Carle, R. Flavonol Glycosides from Distilled Petals of Rosa damascena Mill. *Z. Nat. C***2005**, *60*, 379–384, doi:10.1515/znc-2005-5-602.
99. Chen, Y.; Zhao, Y.; Hu, Y.; Wang, L.; Ding, Z.; Liu, Y.; Wang, J. Isolation of 5-hydroxypyrrolidin-2-one and other constituents from the young fronds of Pteridium aquilinum. *J. Nat. Med.***2008**, *62*, 358–359, doi:10.1007/s11418-008-0225-4.
100. Bai, N.; He, K.; Roller, M.; Lai, C.-S.; Shao, X.; Pan, M.-H.; Ho, C.-T. Flavonoids and Phenolic Compounds from Rosmarinus officinalis. *J. Agric. Food Chem.***2010**, *58*, 5363–5367, doi:10.1021/jf100332w.
101. Badr, A.M.; El-Demerdash, E.; Khalifa, A.E.; Ghoneim, A.I.; Ayoub, N.A.; Abdel-Naim, A.B. Rubussanctus protects against carbon tetrachloride-induced toxicity in rat isolated hepatocytes: Isolation and characterization of its galloylated flavonoids. *J. Pharm. Pharmacol.* **2009**, *61*, 1511–1520.

102. Chung, S.-K.; Kim, Y.-C.; Takaya, Y.; Terashima, K.; Niwa, M. Novel flavonol glycoside, 7-O-methyl mearnsitrin, from Sageretiatheezans and its antioxidant effect. *J. Agric. Food Chem.* **2004**, *52*, 4664–4668.
103. Schmitzer, V.; Veberic, R.; Slatnar, A.; Stampar, F. Elderberry (Sambucus nigraL.) Wine: A Product Rich in Health Promoting Compounds. *J. Agric. Food Chem.***2010**, *58*, 10143–10146, doi:10.1021/jf102083s.
104. Christopoulou, C.; Graikou, K.; Chinou, I. Chemosystematic Value of Chemical Constituents fromScabiosahymettia (Dipsacaceae). *Chem. Biodivers.***2008**, *5*, 318–323, doi:10.1002/cbdv.200890029.
105. Wolbiś, M.; Nowak, S.; Kicel, A. Polyphenolic compounds in ScopoliacaucasicaKolesn. ex Kreyer (Solanaceae). *Acta Pol. Pharm.* **2007**, *64*, 241–246.
106. Nowak, S.; Wolbiś, M. Flavonoids from some species of genus Scopolia Jacq. *Acta Pol. Pharm.* 2002, *59*, 275–280.
107. Çalis, I.; Zor, M.; Basaran, A.A.; Wright, A.D.; Sticher, O. Karsoside and scropolioside D, two new iridoid glycosides from Scrophulariailwensis. *J. Nat. Prod.* **1993**, *56*, 606–609.
108. Wang, W.-S.; Lu, P.; Duan, C.-H.; Feng, J.-C. A new jacaranone derivative from Senecio scandens var. incisus. *Nat. Prod. Res.***2010**, *24*, 370–374, doi:10.1080/14786410903250936.
109. Huang, H.-C.; Syu, K.-Y.; Lin, J.-K. Chemical Composition of Solanum nigrum Linn Extract and Induction of Autophagy by Leaf Water Extract and Its Major Flavonoids in AU565 Breast Cancer Cells. *J. Agric. Food Chem.***2010**, *58*, 8699–8708, doi:10.1021/jf101003v.
110. Wu, B.; Takahashi, T.; Kashiwagi, T.; Tebayashi, S.-I.; Kim, C.-S. New flavonoid glycosides from the leaves of Solidagoaltissima. *Chem. Pharm. Bull.* **2007**, *55*, 815–816.
111. Choudhary, M.I.; Naheed, N.; Abbaskhan, A.; Ali, S. Hemiterpene glucosides and other constituents from Spiraea canescens. *Phytochemistry* **2009**, *70*, 1467–1473, doi:10.1016/j.phytochem.2009.07.013.
112. Cai, L.; Wu, C.D. Compounds from Syzygiumaromaticum possessing growth inhibitory activity against oral pathogens. *J. Nat. Prod.* **1996**, *59*, 987–990.
113. Abouzid, S.F.; Ali, S.A.; Choudhary, M.I. A new ferulic acid ester and other constituents from Tamarixnilotica leaves. *Chem. Pharm. Bull.* **2009**, *57*, 740–742.
114. Marzouk, M.S.; El-Toumy, S.A.; Moharram, F.A. Pharmacologically Active Ellagitannins from Terminalia myriocarpa. *Planta Med.***2002**, *68*, 523–527, doi:10.1055/s-2002-32549.
115. Abe, F.; Iwase, Y.; Yamauchi, T.; Yahara, S.; Nohara, T. Flavonolsinapoyl glycosides from leaves of Thevetia peruviana. *Phytochemistry* **1995**, *40*, 577–581.
116. Sharaf, M. Chemical constituents from the seeds of Trifoliumalexandrinum. *Nat. Prod. Res.* **2008**, *22*, 1620–1623.
117. Han, Y.; Nishibe, S.; Noguchi, Y.; Jin, Z. Flavonol glycosides from the stems of Trigonella foenum-graecum. *Phytochemistry***2001**, *58*, 577–580, doi:10.1016/s0031-9422(01)00273-4.
118. Dini, A.; Rastrelli, L.; Saturnino, P.; Schettino, O. Minor components in food plants--Note I. Flavonol glycosides from Ullucustuberosus. *Boll. Della Soc. Biol. Sper.* **1991**, *67*, 1053–1058.
119. Ek, S.; Kartimo, H.; Mattila, S.; Tolonen, A. Characterization of Phenolic Compounds from Lingonberry (Vaccinium vitis-idaea). *J. Agric. Food Chem.***2006**, *54*, 9834–9842, doi:10.1021/jf0623687.
120. Majinda, R.R.; Motswaledi, M.; Waigh, R.D.; Waterman, P.G. Phenolic and antibacterial constituents of Vahliacapensis. *Planta Med.***1997**, *63*, 268–270.
121. Malafronte, N.; Pesca, M.S.; Bisio, A.; Escobar, L.M.; De Tommasi, N. New flavonoid glycosides from Vernonia ferruginea. *Nat. Prod. Commun.***2009**, *4*, 1934578X0900401205, doi:10.1177/1934578x0900401205.
122. Seetharaman, T.; Petrus, A. Note: Novel acylkaempferol glycoside from the endemic species, Vernonia travancorica Hook. f. *J. Asian Nat. Prod. Res.***2004**, *6*, 295–299, doi:10.1080/1028602031000147401.
123. Szostak, H.; Kowalewski, Z. The flavonoids in the leaves of Vinca minor L. (Apocynaceae). *Pol. J. Pharmacol. Pharm.***1975**, *27*, 657–663.
124. Castillo-Muñoz, N.; Gómez-Alonso, S.; García-Romero, E.; Hermosín-Gutíerrez, I. Flavonol Profiles of VitisviniferaRed Grapes and Their Single-Cultivar Wines. *J. Agric. Food Chem.***2007**, *55*, 992–1002, doi:10.1021/jf062800k.
125. Rao, Y.K.; Fang, S.-H.; Tzeng, Y.-M. Inhibitory effects of the flavonoids isolated from Waltheriaindica on the production of NO, TNF-α and IL-12 in activated macrophages. *Biol. Pharm. Bull.* **2005**, *28*, 912–915.

126. Manguro, L.O.A.; Ugi, I.; Lemmen, P.; Hermann, R. Flavonol glycosides of Warburgiaugandensis leaves. *Phytochemistry* **2003**, *64*, 891–896.
127. Niklas, K.J.; Giannasi, D.E. Flavonoids and Other Chemical Constituents of Fossil Miocene Zelkova (Ulmaceae). *Science* **1977**, *196*, 877–878, doi:10.1126/science.196.4292.877.
128. Coelho, R.G.; Di Stasi, L.C.; Vilegas, W. Chemical constituents from the infusion of ZollerniailicifoliaVog. and comparison with Maytenus species. *Z. Nat. C* **2003**, *58*, 47–52.
